# Niemann-Pick Disease: A Case Report and Literature Review

**DOI:** 10.7759/cureus.33534

**Published:** 2023-01-09

**Authors:** Paola Jacqueline Vélez Pinos, Michell Susan Saavedra Palacios, Paolo Andrés Colina Arteaga, Tania Diciana Arevalo Cordova

**Affiliations:** 1 Pediatrics, University of Azuay, Cuenca, ECU; 2 College of Medicine, University of Cuenca, Cuenca, ECU; 3 College of Medicine, Catholic University of Santiago de Guayaquil, Guayaquil, ECU; 4 Medicine, Technical University of Machala, Machala, ECU

**Keywords:** inborn errors, metabolism, lysosomal storage diseases, sphingolipidoses, niemann-pick disease

## Abstract

Niemann-Pick disease (NPD) A/B is a lysosomal storage disease (LSD), caused by an autosomal recessive disorder that causes variation in sphingomyelin phosphodiesterase-1 (SMPD1). Systemic signs are cholestatic jaundice in the neonatal period or hepatosplenomegaly in infancy. The clinical course experienced by our patient did not correspond to the classic phenotypes. The diagnosis was effectively made at four years and three months of age when different signs such as abdominal distension, hepatosplenomegaly, and chronic malnutrition were present. Given the high suspicion of metabolic storage disease, an enzyme activity study, liver and bone marrow biopsies, and molecular studies were performed. In the bone marrow biopsy, pseudo-Gaucher foam cells were observed. Additionally, the liver biopsy showed dispersed ballooned cells with deposit material and nested cells with granular material. The double enzymatic assay was ordered to determine if the cause of these findings was due to Niemann-Pick or Gaucher disease; decreased sphingomyelinase activity values were obtained (0.28 mcoml/L/h). Subsequently, the molecular genetics study reported a double alteration in the sequence that encodes the SMPD1 gene, located on chromosome 11p15.4, which confirmed NPD type A or B. The overlap and the lack of some findings made the diagnosis very difficult. Diagnosis is crucial due to the multisystem involvement that this LSD can have.

## Introduction

According to the report of 2021 in the portal of rare diseases and orphan drugs (Orphanet), it was estimated that more than 300 million people in the world have some pathology that is considered rare [1]. Among these, lysosomal storage diseases (LSDs), comprise a group of at least 50 distinct genetic diseases, each one resulting from a deficiency of a particular lysosomal protein/activity or, in a few cases, from non-lysosomal activities that are involved in lysosomal biogenesis or protein maturation. The estimated overall prevalence is one in 7,700 live births with a high prevalence in the Ashkenazi population. There is no information on the disease's prevalence in Ecuador, Mexico, etc., but in Chile, the p.Ala359Asp mutation that causes Niemann-Pick type B has been reported to have a carrier frequency of 1:105, indicating a disease incidence of 1:44,960 [2]. Reported epidemiological data is probably underestimated due to the misdiagnosis of these rare disorders and directly influences the impact of LSDs, such as Gaucher, Fabry, and Niemann Pick, among others in the community [3]. 

Niemann-Pick disease (NPD) is an LSD caused by an autosomal recessive disorder resulting from variants in the sphingomyelin phosphodiesterase-1 (SMPD1) gene. In 1914, this disease was described clinically for the first time by the pediatrician Albert Niemann and in 1927, the pathologist Ludwig Pick made its histological description. Later in 1961 and 1966, the separate works of Allen Crocker and Roscoe Brady classified it into four types: A, B, C, and D in which deficiencies of lysosomal proteins cause improper intracellular lipid trafficking causing neurological impairment and death [2,4]. NPD is currently divided into two distinct entities, the first one is acid sphingomyelinase-deficient NPD (ASM-deficient NPD) resulting from mutations in the SMPD1 gene and encompassing type A and type B as well as intermediate forms; the second one is NPD type C (NPC) also including type D, resulting from mutations in either the NPC1 or the NPC2 gene which are currently described as a cellular cholesterol trafficking defect but in the brain, the prominently stored lipids are gangliosides, those that present different signs-symptoms, depending on the type of mutation they present [5].

The systemic signs are cholestatic jaundice in the neonatal period or hepatosplenomegaly in childhood. They also depend on the age of onset, early infantile period (delay in developmental motor milestones), late infantile and juvenile period (gait problems, falls, clumsiness, cataplexy, school problems), and adult period (ataxia and psychiatric disturbances); the majority of the patients affected may present with vertical supranuclear gaze palsy, cerebellar ataxia, dysarthria, dysphagia, and progressive dementia [5]. 

The importance of clinical case presentations helps to internalize information about rare diseases such as NPD. Therefore, when a pediatric case presents with hepatomegaly or splenomegaly, even if they are not pathognomonic signs, the experienced physician can suspect this type of pathology and request tests to confirm or rule out common diseases. This paves the way for prompt diagnosis and intervention before the occurrence of serious and irreversible manifestations, thereby providing substantial benefit to both affected newborns and their parents.

## Case presentation

Our patients' perinatal history was as follows: male newborn, normal childbirth, weight suitable for gestational age (weight: 2800 g, size: 48 cm, head circumference: 33 cm, Apgar: 8-9, Capurro: 39 weeks gestation); the physical examination was normal. During the pregnancy, the mother had no abnormalities, no comorbidities, and no history of exposure to toxic agents. None of her family members had been affected by a degenerative, chronic, hereditary disease or died unexpectedly.

At two months of age, our patient was hospitalized for bronchiolitis; he presented a low stature of 58 cm (Z-score 0.14) and a low weight of 4300 g (Z-score -1.14). He was readmitted at 18 months due to the diagnosis of moderate pneumonia, without other evident abnormal physical findings. At the time, he had a weight of 10200 g (Z-score -0.87) and a stature of 75 cm (Z-score -2.38). At two years and 10 months of age, he was referred to the pediatric outpatient clinic of a second-level hospital due to chronic malnutrition; he presented with a weight of 13000 g (28.6 lb.) (Z-score -0.61), a height of 85 cm (Z-score -2.61), BMI of 18.1 (Z-score 1.46); splenomegaly was also evident. Hematology evaluation was requested but the mother was not present.

**Figure 1:**
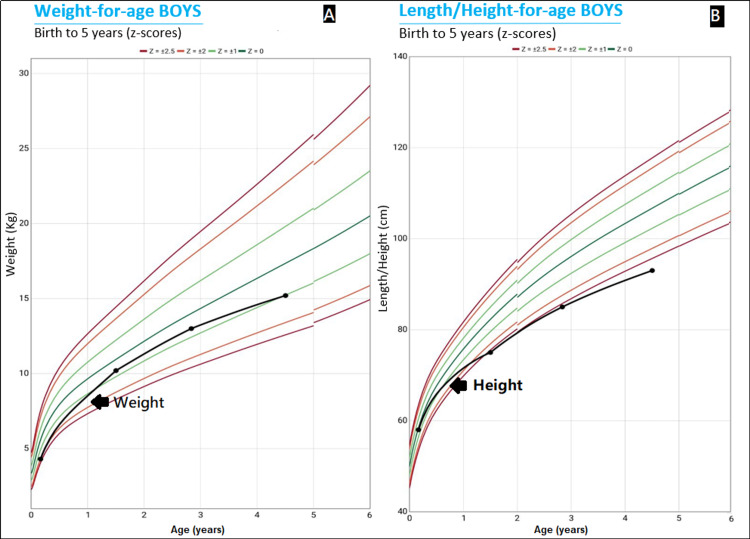
WHO child growth standards A. Weight report for the patient; B. Height report for the patient.

At four years and three months, due to abdominal distension, the patient was transferred from a first-level type A health center to an outpatient clinic at a second-level hospital. Anthropometric data at the time was as follows: weight of 15.2 kg (Z-score -1.12), height of 93 cm (Z-score -3), and BMI of 17.6 (Z-score +1.31). The Child Growth Curve for Weight and Height is presented in Figure 1. Physical examination revealed non-tender abdominal distension, hepatomegaly 3 cm below the costal margin with regular borders, and splenomegaly with the tip of the spleen 10 cm below the costal margin (grade 2) which was painless on superficial and deep palpation. Laboratory tests were performed where complete blood count, electrolytes, coagulation times, lipid profile, and iron profile report values ​​were within the normal range (Table 1). Abnormal lab values were seen in the liver function test (Table 2). Serology of viral markers revealed positive IgG Ab for herpes simplex virus type 1 and negative RT-PCR testing for SARS-CoV-2.

**Table 1:** Laboratory tests including complete blood count, electrolytes, coagulation times, lipid profile, and iron profile reports Hb: hemoglobin; PT: prothrombin time; PTT: partial thromboplastin time; INR: international normalized ratio; HDL-C: high-density lipoprotein cholesterol; LDL-C: low-density lipoprotein cholesterol; VLDL-C: very low-density lipoprotein cholesterol. * Pediatric normal laboratory values, Department of Anesthesiology and Pediatrics, Baylor College of Medicine, and Texas Children’s Hospital, Houston, TX, USA.

	Lab values	Normal values*.
Complete blood count		
Hb., g/dl	14.7	11.5 - 14.5
Leukocyte count, 10^3/uI	13.26	5.0 - 14.5
Platelets, 10^3/uI	279.00	150.00 - 450.00
Electrolytes		
Sodium, mmol/L	145	136 - 145
Potassium, mmol/L	4.4	3.5 - 5.5
Chloride, mmol/L	108	95 - 105
Iron studies		
Iron, μg/dL	102.7	55 - 150
Ferritin, ng/mL	61.55	36 - 84
Transferrin, mg/dl	224	169 - 300
Transferrin saturation, %	45.8	15 - 39
Coagulation time		
PT	13.0	12.2 - 15.5
PTT	34.9	26.5 - 35.5
INR	1.1	0.8 - 1.2
Lipid Profile		
Total Cholesterol, mg/dl	170.8	135 - 200
HDL - C, mg/dl	28.1	38 - 75
LDL - C, mg/dl	114	64 - 130
VLDL - C	28.8	
Triglycerides, mg/dl	144.0	20 - 150

**Table 2:** Liver function test reports AST: aspartate aminotransferase; ALT: alanine aminotransferase. * Pediatric normal laboratory values, Department of Anesthesiology and Pediatrics, Baylor College of Medicine, and Texas Children’s Hospital, Houston, TX, USA.

	Starting lab values (March 4, 2020)	Ending lab values (October 6, 2021)	Normal values*.
AST, U/L	41.0	61.6	15-50
ALT, U/L	21.4	40.3	10-25
Total bilirubin, mg/dl	0.95	1.52	0.2 - 1.0
Direct bilirubin, mg/dl	0.27	0.29	<0.35

The portal Doppler ultrasound revealed an increased size of the right lobe of the liver (12.2 mm) and increased size of the spleen (11.5 cm x 5 cm on its largest diameters), concluding the diagnosis of hepatosplenomegaly. The patient was assessed by a neurologist who reported no pathological findings. Additionally, the electroencephalogram studies and computed tomography of the head did not present any abnormalities.

**Figure 2:**
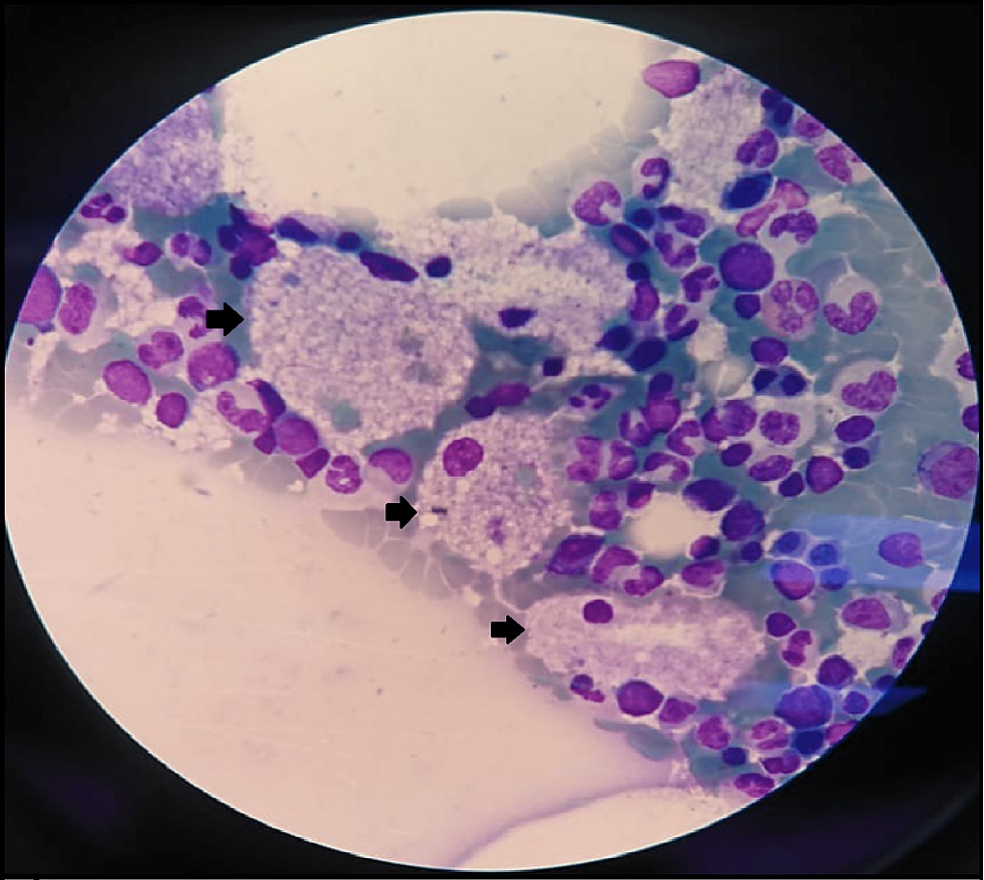
Bone marrow biopsy Normal bone marrow cells are seen. Arrows indicate foam cells consistent with Niemann-Pick disease (H&E, 400x).

**Figure 3:**
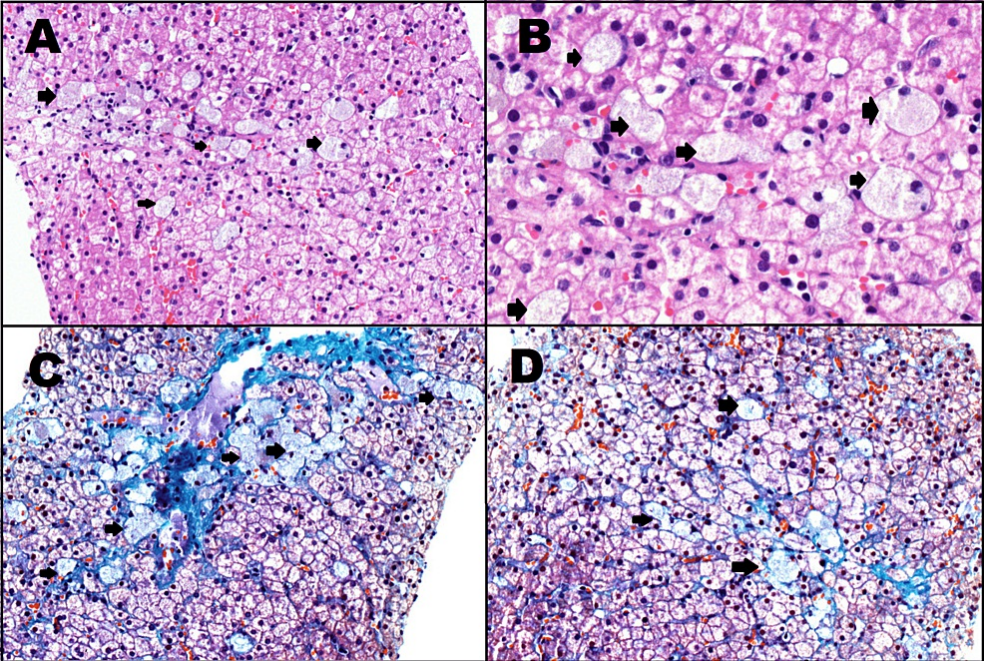
Liver biopsy A,B. Disperse ballooning cells with deposit material (H&E A: 200x, B: 400x). C,D. Disperse cell nesting with fine granular material (Masson trichrome stain, 200x).

**Figure 4:**
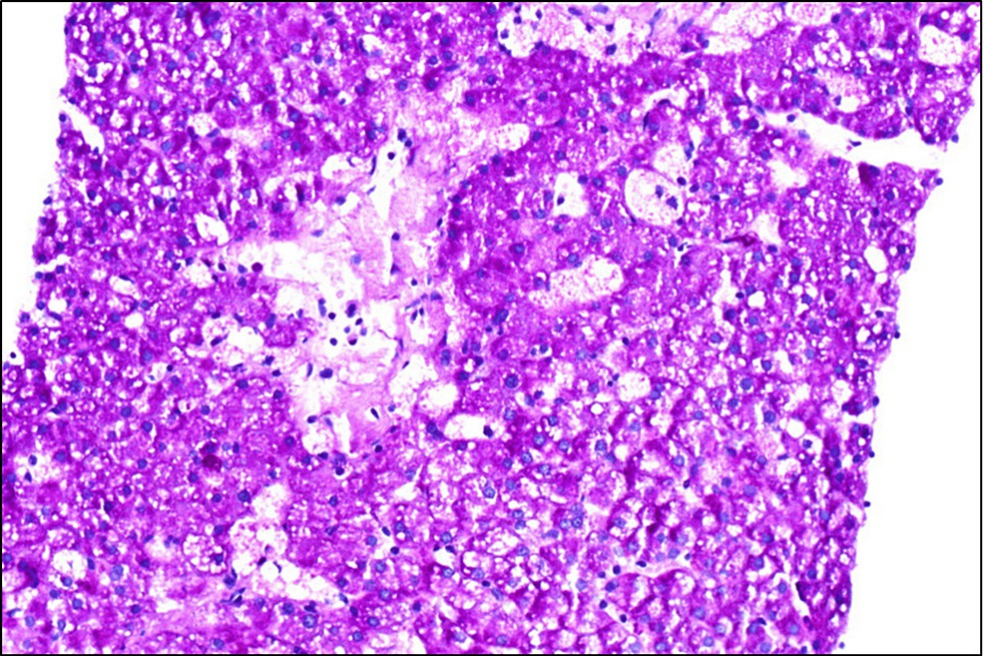
Liver biopsy Periodic acid-Schiff (PAS) stain (200x).

After a thorough review of imaging and laboratory studies, infectious, hematological, and tumoral pathologies were ruled out. After a medical meeting, a decision was made to perform enzyme activity assay, liver and bone marrow biopsies, and molecular testing due to high suspicion of metabolic storage disease. Bone marrow biopsy was performed through the left internal iliac crest (1 cm) where pseudo-Gaucher foam cells were observed (Table 3, Figure 2). Additionally, liver biopsy showed dispersed ballooning cells with deposit material and nested cells with granular material (Figures 3-4).

**Table 3:** Bone marrow and tissue biopsy H&E: haematoxylin and eosin stain; HPF: high power field; RBCs: red blood cells; WBCs: white blood cells.

Report	
Cellularity according to age, infiltration of foamy cells with eccentric nuclei and abundant cytoplasm	3-4 cells/HPF
RBCs	normal appearance and quantity, normoblasts predominance
WBCs	normal appearance
Megakaryocyte	Increased 5-6 cells/HPF

Based on the results of the biopsies performed and to differentiate whether the cause of the findings was NPD or Gaucher disease, a double enzymatic assay was performed, in which decreased sphingomyelinase activity values were obtained (0.28 mcoml/L/h). To complete the enzymatic study, a molecular genetic study was requested which reported a double alteration in the sequence that encodes the SMPD1 gene; it was located on chromosome 11p15.4 which confirmed the diagnosis of NPD type A or B i(Table 4).

**Table 4:** Molecular genetic study Results: abnormal sequencing analysis of SMPD1.

Exon/Intron	Nucleotide change	Amino acid change	Zygosity	Type	Database #
Exon 1	c.28C>T	p.Gln10	Heterozygous	Likely pathogenic	DBSP rs1205990349
Exon 2	c.362T>C	p.Leu121Pro	Heterozygous	Likely pathogenic	HGMD CM162536

During the hospitalization, the patient received supportive treatment for abdominal discomfort and later was discharged with appropriate disease-prognosis counseling. At the moment, the patient’s mother reports occasional mild epistaxis, but platelets and coagulation studies remain normal. The patient keeps attending routine follow-ups and no specific treatment has been given at the moment.

## Discussion

The clinical course that our patient experienced does not correspond to the classic phenotypes [2,4,5]. Diagnostically, the criteria for either type A and B sphingomyelinase deficiency were met [2-5]. The overlapping findings and the lack of pathognomonic signs [6] made the diagnosis very challenging. The protracted clinical course is unquestionably interesting as evidenced by the relatively high degree of tolerance to the effects of enzyme deficiency. There is no simple explanation for the disease tolerance by the patient. The diagnosis was effectively done at four years and three months of age when abdominal distention, hepatosplenomegaly, and chronic malnutrition were found. Although the patient started to manifest signs earlier, the barriers in follow-up delayed the diagnosis. The multisystem build-up inside macrophages of certain lipids that cannot be metabolized due to enzyme deficiency produces irreversible damage and abnormal growth of different organs. Developmental delays, loss of motor skills, and hepatosplenomegaly are the most prominent findings of this disease [6]. 

Our patient presented with painless abdominal distention; imaging revealed hepatosplenomegaly which is the most common sign of NPD type A/B. Its appearance occurs in over 90% of the cases [7]. There is major significance in giving priority to this abnormality because its presentation in childhood can predispose patients to liver failure which is the first cause of death from this disease. In addition, early in adult life, this disease can lead to death mainly from respiratory failure [7]. 

Growth delay has been linked mostly to NPD type B and A/B [8]. There is evidence that the severity of growth delay correlates tightly with decreased insulin growth factor 1 (IGF-1), decreased bone age, and degree of organomegaly. This is partially explained due to liver dysfunction [9]. Although in our case, bone and joint pain were not present, the patient had growth delay which was evident since he was first seen at two months of age; he presented with low height and weight which correlated with NPD type B along with other cardinal signs of the disease [10].

Organomegaly, such as liver enlargement in infancy and childhood, is not uncommon and may require extensive evaluation to distinguish benign, self-limited processes from life-threatening conditions, with a specific emphasis on those conditions such as NPD which necessitates hospital admission and complete evaluation. It is possible to address this finding through the pathophysiological mechanisms that produce it such as inflammation, Kupffer cell hyperplasia, congestion, infiltration, fat accumulation, tumor, or intrinsic mechanism, which helps classify or rule out pathologies. If the findings correspond to a storage mechanism, it leads us to think of an LSD. 

Interstitial lung disease could present with symptoms of respiratory distress, diffuse interstitial pattern on imaging, or a restrictive pattern in the pulmonary function test [12]; none of them were present in our patient. Therefore, regular pulmonary evaluation is recommended to ensure early management and mortality [7].

One-third of individuals with Niemann-Pick B present with ophthalmological findings such as a macular halo or a cherry red spot macula, in contrast to patients with Niemann-Pick A, in which this sign is evidenced at the time of diagnosis in 50% of the patients and will be present in 100% of them by the age of one year [13]. In our case, the patient was diagnosed with Niemann-Pick A/B, so its phenotype may belong to two-thirds of Niemann-Pick B patients who do not present with this manifestation. Nevertheless, due to its genotype, it is possible that in the future, this alteration becomes evident with the progression of the disease.

Neurologic manifestations predominantly appear in NPD type A and are excluded in type B, often presenting with hypotonia, seizures, and loss of deep tendon reflexes [14]. Brain imaging is usually normal but it may show leukodystrophy, white matter signal hyperintensity in T2, or brain atrophy, which are the characteristic findings [6]. The absence of these findings do not rule out the probability that our patient may present neurological manifestations later, due to the heterogeneity of his disease. Currently, developmental milestones are normal and show no regression.

A finding currently described and evident from the earliest age that contributes to cardiac disease (due to atherosclerosis) is lipid abnormalities, usually characterized by low serum concentrations of HDL-C accompanied by hyperlipidemia which is characterized by hypertriglyceridemia and elevated levels of LDL-C and VLDL-C [15]. Our patient presented values within the upper limit, with the probability of having altered laboratory values over time.

Histopathological examination of the bone marrow and biopsy of commonly involved organs such as the liver, spleen, and intestine could be an important clue that suggests NPD. The biopsy findings in our patient were decisive enough to continue with the diagnostic approach, even when they are not pathognomonic. The common findings are foamy cells up to 90 microns with an eccentric, small, round nucleus, and irregular vacuoles that may be mulberry or soap bubble-like, positive for lipid stain (Sudan Black) and negative for Periodic acid-Schiff (PAS) stain [2]. If this disease is suspected, the next step would be liver and bone marrow biopsies, in which the presence of lipid-laden macrophages (foam cells) in the bone marrow, spleen, and liver is the clue for the diagnosis, but it should not substitute the need for enzyme activity tests [16].

It is important to mention that since the target organs for the deposit of the disease are the liver, spleen, and bone marrow, laboratory findings are important for diagnostic support. Our patient did not present cytopenias to date which is in contrast to several clinical laboratory studies; the studies revealed that patients with NPD commonly have thrombocytopenia (>50% of patients), anemia, and leukemia (affects approximately 20%-30% of patients) [4,7,15]. It is documented that thrombocytopenia and leukopenia tend to increase with the patient’s age [8]. Recently, our patient has started to present epistaxis, a clinical manifestation that can occur in 29% of patients. The pathophysiology is not entirely clear, but it is suspected to be related to thrombocytopenia [15]. On the other hand, another study correlates it to the degree of splenomegaly or platelet dysfunction [8]. General laboratory studies that should be performed include liver function tests such as alanine transaminase (ALT), aspartate transaminase (AST), alkaline phosphatase (ALP), albumin and total protein, as well as gamma-glutamyltransferase (GGT), L-lactate dehydrogenase (LD), etc. [11]. At the time of hospitalization, our patient presented values within the upper limit of AST and ALT, which gradually increased.

The measuring of sphingomyelinase activity (using sphingomyelinase substrate in white blood cells or cultured skin fibroblasts) includes or excludes the diagnosis sphingomyelinase disease. After that, DNA extraction and sequencing are performed for the mutational analysis of the SMPD1 gene. Due to its high specificity, it is the gold standard to confirm the diagnosis; however, it should never be the initial test [14,17].

A research article about the genotype-phenotype correlations in patients with NPD type A/B described longer survival means in patients with slow neurological involvement [4]. Our patient presented with genetic results of the SMPD1 gene, where he had c.28C>T (p.Gln10) in exon 1, and c.362T>C (p.Leu121Pro) in exon 2. Heterozygous mutations, such as this one, have been documented with residual enzyme activity that correlates with less severe neurological signs. This shows that the documented variant may not be the only one associated with the mild and protracted presentation of the disease. The occurrence of genetic heterogeneity resulting in dramatically different phenotypes is a hallmark of LSDs. The identification of additional mutations causing types A and B NPD should permit reliable genotype/phenotype correlations and provide further insights into the functional organization of the acid sphingomyelinase polypeptide [18].

As of now, NPD type A/B is not curable. No disease-modifying treatments have been approved by the United States Food and Drug Administration (FDA). However, a multidisciplinary approach can address symptomatic management through supportive measures and palliation [15]. In our case, the patient required an interprofessional team; now he continues to be on on track with his primary care physician. 

As of now, enzyme replacement therapy (ERT) with recombinant human acid sphingomyelinase (rhASM) is currently being studied as a potentially disease-modifying therapeutic for the treatment of (rhASM) [15,19]. Recently Olipudase alfa, a specific ERT, demonstrated safety and efficacy in treating the non-neurological manifestations of rhASM. On the other hand, other approaches that are being studied are gene therapy [19] and intravenous (IV) trehalose [20].

Early diagnosis is crucial for the prompt management of life-threatening symptoms and to prevent early mortality. Due to the rarity and the heterogeneity of the disease, the diagnosis can be delayed or lost; suspicioun is raised when the patient presents pathognomonic symptoms that become evident at an advanced stage of the disease where enzymatic and genetic testing is required for confirmation [2,15]. Only further research will show whether neurological and visceral involvement is indeed the relevant distinguishing trait in the different types of NPD. In addition, it is important to highlight that in Latin America, this disease is underdiagnosed [2,3], and it is hard to find real statistics that show its impact. 

The patient must be kept under medical follow-up since acid sphingomyelinase deficiency (ASMD) is a progressive disease that often develops complications with time. Some of the complication are hepatic failure, respiratory insufficiency, dementia, seizures, and schizophrenia-like psychosis due to progressive neurodegeneration, profuse bleeding (internal/external), coronary artery and valvular heart disease, and osteopenia that could cause bones deformities ending in enlarged bone marrow cavities, thinned cortical bone or cosa vara [13].

The prognostic of Niemann-Pick A is almost always fatal; type B has a slightly better prognosis and patients may live till late childhood or early adulthood; in type C, the survival time is not beyond five years of age, and if it persists after five years, patients may live to the age of 20 [13]. Our patient, who has NPD A-B, manifested a non‐neuronopathic clinical form of NPB [4] where the disease adopts a chronic course. 

## Conclusions

The clinical course that our patient experienced does not correspond to the classic phenotypes. Diagnostically, the criteria for either type A and B sphingomyelinase deficiency were met. The overlapping findings and the lack of pathognomonic signs of the disease made the diagnosis very challenging. The prolonged clinical course of the disease was undoubtedly interesting due to our patient's relatively high degree of tolerance to the effects of this enzyme deficiency, which presupposes the absence of neurological, pneumatological, and haematological signs. 

Diagnosis is crucial due to the multisystemic involvement that this LSD carries. This makes early diagnosis crucial; prompt management of life-threatening symptoms can prevent early mortality. Due to the rarity and heterogeneity of the disease, the diagnosis may be missed or delayed; it is usually suspected in the final stages when symptomatology is evident, requiring further enzymatic and genetic testing. Through our case report, we aim to provide visibility and awareness to primary care physicians about the signs they should recognize for an early and correct diagnosis. It will also help them with prompt management of life-threatening symptoms to prevent early mortality from any complications.
